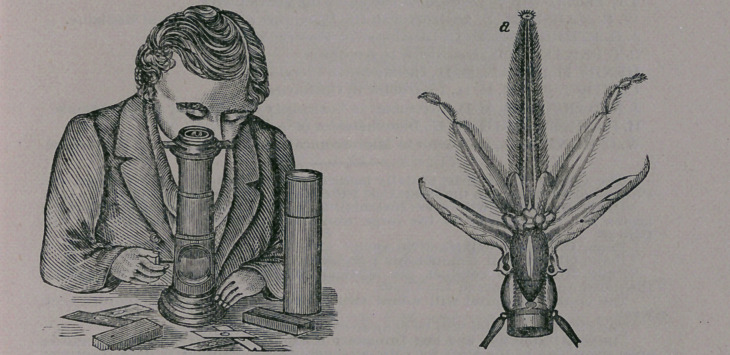# Items

**Published:** 1869-03-01

**Authors:** 


### *Items.*

Difficult and Tedious Labor—With inertia of the uterus and rigidity of the *os*, especially in primiparous cases over thirty years of age, is treated by our friend J. W. Dora, M. D., of Mattoon, as follows:

"After having administered either chloroform by inhalation or having applied ung: belladonnæ to the *os* for the purpose of promoting dilitation of the same, I give, viz:

Fld ext. of Ergott ℥ j.

Chloroform ℥ ss.

Syr. of Acacia " "

M. Sig. Teaspoonful every 20 or 30 minutes until the labor is well established, which I have never known to fail after the third dose, and repeat at such intervals as may be indicated afterwards. It has never disappointed me, and I never knew any unpleasant results from its use, either to the mother or child. J. W. Dora."

The Craig Microscope.—This little instrument, afforded at the trivial price of $2.75 (See our advertising sheet), is really a very clever affair. It magnifies, we should judge, from 80 to 100 diameters, and is easily adjusted. In the absence of more expensive instruments it can be employed very satisfactorily in the study of the minute anatomy of tissues, and in the examination of the epithelium in cutaneous diseases, the blood, pus, mucous, urine, etc. A couple of cuts are subjoined which illustrate the instrument and its mode of use, and, also, its exhibition of a bee's tongue.

THE Summer Course of Examinations and Lectures in connection with the Summer Lectures at the Jefferson Medical College, will begin on Tuesday, April 13, 1869, at 8.45 o'clock, A. M., at the Philadeldhia School of Anatomy, Chant street, (Tenth street above Chestnut.)

The examinations will be held daily, excepting Wednesday and Saturday, and will have direct bearing on the lectures and clinics. Lectures will also be delivered by Dr. Hutchins on Diseases of Women, with clinical facilities to the class, and by Dr. Allis on Physiology. The examinations and lectures will be illustrated by diagrams, models, cabinet of materia medica, etc. Office students will be taken by either members of the Association.

Dr. Keen, Anatomy and Chemistry; Dr. Hutchins, Obstetrics, Practice and Materia Medica; Dr. Allis, Surgery and Physiology.

Fee for the Examinations, $20. Fee for Office Students, (one year, including Winter and Summer Examinations,) $100. For further information apply at the Rooms, or to W. W. KEEN, M. D., 1619 Chestnut Street.
E. R. HUTCHINS, M. D., 269 So. 5th Street.
O. H. ALLIS, M. D., 221 So. 9th Street.

LITHOGRAPH.—Mr. CHAS. KEIL, Janitor of Rush Medical College, has had engraved, from photographs, a sheet containing the portraits of the present Faculty of the College. In the main the likenesscs are very good. Copies will be mailed postpaid to those desiring them, by enclosing $2.00 and addressing, CHARLES KEIL,
Rush Medical College, Chicago.

***Special Notice to Subscribers.***

Upon the books of THE JOURNAL appear the names of a large number of subscribers who are in arrears for subscriptions. We have had an immensity of trouble in correspondence about this thing, and wish to have everything cleared up at once. To any subscriber who is in arrears we propose this: Pay at once what you think you owe, and so state, and we will balance your account on our books. We are tired and disgusted at looking over the old ledger, and have ordered a new one which we mean shall be a *clean* record.

## Figures and Tables

**Figure f1:**